# Comparing short versions of the AUDIT in a community-based survey of young people

**DOI:** 10.1186/1471-2458-13-301

**Published:** 2013-04-04

**Authors:** Anna L Bowring, Maelenn Gouillou, Margaret Hellard, Paul Dietze

**Affiliations:** 1 Centre for Population Health, Burnet Institute, 85 Commercial Rd, Melbourne, VIC, 3004, Australia; 2 Department of Epidemiology and Preventive Medicine, Monash University, Melbourne, VIC, Australia; 3 The Nossal Institute for Global Health, The University of Melbourne, Melbourne, VIC, Australia

**Keywords:** Alcohol screening, Alcohol use disorders identification test, AUDIT, Alcohol consumption, Young adult, Adolescents

## Abstract

**Background:**

The 10-item Alcohol Use Disorders Identification Test (AUDIT-10) is commonly used to monitor harmful alcohol consumption among high-risk groups, including young people. However, time and space constraints have generated interest for shortened versions. Commonly used variations are the AUDIT-C (three questions) and the Fast Alcohol Screening Test (FAST) (four questions), but their utility in screening young people in non-clinical settings has received little attention.

**Methods:**

We examined the performance of established and novel shortened versions of the AUDIT in relation to the full AUDIT-10 in a community-based survey of young people (16–29 years) attending a music festival in Melbourne, Australia (January 2010).

Among those reporting drinking alcohol in the previous 12 months, the following statistics were systematically assessed for all possible combinations of three or four AUDIT items and established AUDIT variations: Cronbach’s alpha (internal consistency), variance explained (R^2^) and Pearson’s correlation coefficient (concurrent validity). For our purposes, novel shortened AUDIT versions considered were required to represent all three AUDIT domains and include item 9 on alcohol-related injury.

**Results:**

We recruited 640 participants (68% female) reporting drinking in the previous 12 months. Median AUDIT-10 score was 10 in males and 9 in females, and 127 (20%) were classified as having at least high-level alcohol problems according to WHO classification.

The FAST scored consistently high across statistical measures; it explained 85.6% of variance in AUDIT-10, correlation with AUDIT-10 was 0.92, and Cronbach’s alpha was 0.66. A number of novel four-item AUDIT variations scored similarly high. Comparatively, the AUDIT-C scored substantially lower on all measures except internal consistency.

**Conclusions:**

Numerous abbreviated variations of the AUDIT may be a suitable alternative to the AUDIT-10 for classifying high-level alcohol problems in a community-based population of young Australians. Four-item AUDIT variations scored more consistently high across all evaluated statistics compared to three-item combinations. Novel AUDIT versions may be more effective than many established shortened versions as an alternative screening tool to the AUDIT-10 to measure hazardous or harmful alcohol consumption in this population.

## Background

Risky alcohol consumption is common among Australian young people and associated with elevated incidence of accidents, physical injury and other short-term harm, as well as contributing to long-term health conditions and alcohol dependence [1-4]. In epidemiological research, monitoring alcohol consumption and related harm is important to identify high-risk groups, risk behaviours, and trends in alcohol use in order to inform, target and evaluate harm reduction strategies.

The Alcohol Use Disorders Identification Test (AUDIT) was developed by the World Health Organisation (WHO) as a simple clinical screening tool to detect hazardous and harmful alcohol use and facilitate early intervention in primary care [5]. The AUDIT comprises ten questions covering the domains of alcohol consumption (questions 1–3), alcohol dependence (Q4-6), and harms (Q7-10) and is scored out of 40 [6]. Although the AUDIT was developed as a tool for use in clinical practice, it has been used more broadly for web-based screening and epidemiological research [7-9]. The AUDIT has also been adapted into shortened versions containing subsets of AUDIT items for use in settings subject to time constraints [9]. The most common shortened versions are the AUDIT-C [10], AUDIT-3[11], AUDIT-4 [12], and the Fast Alcohol Screening Test (FAST) [13]). Previous research suggests that shortened versions may be sufficient proxies for the full AUDIT (AUDIT-10), but evaluations of both the AUDIT-10 and shortened AUDIT versions are generally limited to clinical [10-12,14-18] or adult general population samples [7,8,19-21], with the exception of a number of studies of the AUDIT-10 in college students [22-25]. Although some of these studies have reported results among young adults [19,26], there is a need for further research to determine the performance of shortened AUDIT versions in high-risk populations such as young people aged less than 18 years.

We have been conducting alcohol, other drug and sexual risk behaviour surveillance of young people attending a music festival in Melbourne, Australia since 2005 [27,28]. While the AUDIT-10 is a candidate instrument to classify alcohol consumption and risk, space constraints on our questionnaire make it difficult to conduct the full AUDIT-10; a shortened version that captures important information across the consumption, dependence and harms domains suited to our recruitment setting is required; to our knowledge, no such instrument exists. In this study we assess how established and novel shortened versions of the AUDIT perform in relation to the full AUDIT-10 in a community-based sample of young people attending a music festival. Our results will inform our subsequent community-based surveys and be useful for others conducting research with young non-clinical populations.

## Methods

### Setting and recruitment

Individuals aged 16–29 years were recruited at the Melbourne Big Day Out music festival in January 2010 as part of an ongoing behavioural surveillance system, which has been described in detail elsewhere [27-29]. The one-day music festival had over 50,000 attendees in 2010 [30], but the proportion of attendees aged 16–29 is unknown. In brief, approximately 20 trained researchers recruited participants from in and around a market stall within the festival and explained the survey and involvement. Participants were asked to self-complete a consent form and two-page questionnaire. We provided participants with educational materials on alcohol and drug use, sexual health, and mental health, and condoms; participants were also offered bottled water, lollipops, and entry to a prize draw as an incentive for participation.

### Questionnaire

Each year study participants are asked a core set of questions including demographics, sexual health and behaviour, alcohol consumption and other drug use [27,28,31]. In 2010 we used the AUDIT-10 to assess alcohol consumption and risk.

### Analysis

Data were entered into a Microsoft Access database and statistical analysis was conducted in Stata version 11 [32]. The analysis excluded participants who reported never drinking in the past 12 months (n = 34) or who had missing responses to any AUDIT items (n = 17). The AUDIT-10 was scored from 1 to 40 [6]. Additionally, established shortened versions of the AUDIT were scored (Table 1). AUDIT-10 scores were further classified into hazardous drinking (≥8), high-level of alcohol problems (≥16), and possible alcohol dependence (≥20) categories according to WHO recommended thresholds [6]. Comparisons of these classifications by sex were assessed using the *χ*^2^ test of proportions.

**Table 1:** Explained variance of individual AUDIT items to AUDIT-10 score and item inclusion in common established shortened AUDIT versions

				**Established AUDIT Variations**				
**Item no.**	**The AUDIT questions**	**AUDIT Domain**^**1**^	**Explained variance of total AUDIT-10 score (R**^**2**^**)**	**AUDIT-10**	**AUDIT-C**	**AUDIT-3**	**FAST**	**AUDIT-4**
1	How often do you have a drink containing alcohol?	C	30.7%	■	■			■
2	How many drinks containing alcohol do you have on a typical day when you are drinking?	C	30.0%	■	■			■
3	How often do you have 6 or more drinks on one occasion?	C	47.1%	■	■	■	■	■
4	How often during the last year have you found that you were not able to stop drinking once you had started?	D	48.8%	■				
5	How often during the last year have you failed to do what was normally expected of you because of alcohol?	D	44.6%	■			■	
6	How often during the last year have you needed a first drink in the morning to get yourself going after a heavy drinking session?	D	31.3%	■				
7	How often during the last year have you had a feeling of guilt or remorse after drinking?	H	39.6%	■				
8	How often during the last year have you been unable to remember what happened the night before because of your drinking?	H	45.5%	■			■	
9	Have you or someone else been injured because of your drinking?	H	28.9%	■				
10	Has a relative, friend, doctor, or other health care worker been concerned about your drinking or suggested you cut down?	H	34.5%	■			■	■
			**Score range:**	1-40	1-12	0-4	0-16	1-16

^1^ AUDIT domains are: *C*, alcohol consumption; *D*, alcohol dependence; *H*, harmful alcohol use.

All possible combinations of three- and four-item AUDIT scales were evaluated. For our purposes the final shortened AUDIT versions considered were required to: represent all three AUDIT domains (alcohol consumption, C; alcohol dependence, D; and harmful alcohol use, H) and include item 9 on alcohol-related injury, or be an established AUDIT variation with maximum four items (AUDIT-3, AUDIT-C, FAST, AUDIT-4). Item 9 was a key inclusion because of the focus on alcohol-related injury among young people in Australia [33]. Typically, FAST is a four-item abbreviation of the AUDIT but applies different drinking levels for males and females in item 3 (eight and six, respectively) [13]; however, the FAST we used included the standard AUDIT designation of six or more drinks for both sexes.

Three statistics were systematically assessed for all possible combinations of three or four AUDIT items and established AUDIT variations: (1) Cronbach’s alpha was calculated to measure internal consistency of each AUDIT version, with a value of 0.7 or greater taken to indicate satisfactory reliability [5]; (2) R^2^ statistics from linear regression between individual items or novel combination and the total AUDIT score were calculated to measure the total variance explained in the overall AUDIT-10 score by each individual item and novel combination of items; and (3) Pearson’s correlation coefficients were calculated to examine the concurrent validity between each shortened AUDIT version and the AUDIT-10 scale. All shortened AUDIT versions meeting our criteria were ranked from highest to lowest for each of the three statistics, and re-ranked based on the sum of all ranks.

### Ethics

Ethical approval was granted by the Alfred Hospital Human Research Ethics Committee.

## Results

### Demographic profile

Analysis was based on 640 participants completing the AUDIT-10, 68% of whom were female. Participants’ median age was 19.9 years. The majority were Australian-born (93%), lived in a major-city (71%) [34], and lived with parents (64%). Half had completed or were in the process of completing post-high school education. One-fifth of participants reported having used drugs other than alcohol in the month prior to the survey. The median AUDIT-10 score was 10 (interquartile-range [IQR] 7–16) among males and 9 (IQR 6–14) among females. Using WHO recommended threshold scores [6], 417 (65% of the total sample) were classified as at least hazardous drinkers (score ≥8), 127 (20% of the total sample) as having at least high-level alcohol problems (score ≥16), and 65 (10% of the total sample) as possibly alcohol dependent (score ≥20); these classifications did not differ by sex (p = 0.12).

Table 1 shows the individual items of the AUDIT-10 and the items included in common AUDIT variations, along with the variance explained by each item in relation to the total AUDIT-10 score observed in the sample.

In total 330 three- or four- item combinations were possible, and 51 (15%) combinations met the criteria of containing item 9 and representing three domains. An additional four established AUDIT variations were considered, thus eligible AUDIT variations were ranked from 1 to 55 according to performance across the three calculated statistics. Table 2 shows the top ten performing novel combinations of both three and four AUDIT items that meet the study criteria, in addition to the established AUDIT variations.

**Table 2:** Statistical scores of selected novel and established shortened AUDIT variations, ordered from highest to lowest overall ranking

**Overall rank**	**Selected novel & established combinations**	**AUDIT domains**^**1**^	**Explained variance of total AUDIT-10 score (R**^**2**^**)**	**Cronbach’s alpha**	**Pearson’s correlation (r)**
1	FAST (3, 5, 8 & 10)	C, D, H	0.86	0.66	0.92
2	3, 4, 8 & 9	C, D, H	0.87	0.62	0.92
3	3, 4, 5 & 9	C, D, H	0.87	0.61	0.92
4	3, 5, 8 &9	C, D, H	0.87	0.60	0.92
5	3, 4, 7 & 9	C, D, H	0.87	0.59	0.92
6	3, 4, 9 & 10	C, D, H	0.86	0.59	0.91
7	3, 5, 7, 9	C, D, H	0.86	0.59	0.91
8	3, 5, 9 & 10	C, D, H	0.86	0.58	0.90
9.5	3, 4, 6 & 9	C, D, H	0.86	0.55	0.92
9.5	2, 3, 4 & 9	C, D, H	0.83	0.60	0.89
34	3, 4 &9	C, D, H	0.81	0.48	0.88
40	3, 5 & 9	C, D, H	0.80	0.46	0.87
45	AUDIT-4 (1, 2, 3 &10)	C, H	0.70	0.67	0.83
47	AUDIT-C (1, 2 & 3)	C	0.56	0.70	0.74
48	3, 6 & 9	C, D, H	0.78	0.37	0.85
49	1, 4 & 9	C, D, H	0.75	0.43	0.85
50	2, 4 & 9	C, D, H	0.75	0.46	0.85
51	1, 5 & 9	C, D, H	0.74	0.40	0.84
52	2, 5 & 9	C, D, H	0.73	0.44	0.83
53	2, 6 & 9	C, D, H	0.70	0.34	0.81
54	1, 6 & 9	C, D, H	0.68	0.33	0.80
55	AUDIT-3 (3)	C	0.47	NA	0.69

NA- not applicable.

^1^ AUDIT domains are: *C*, alcohol consumption; *D*, alcohol dependence; *H*, harmful alcohol use.

### Cronbach’s alpha (internal consistency)

The internal consistency of the AUDIT-10 in this sample was 0.80. The highest internal consistency of the three-item combinations was obtained with the AUDIT-C (0.70), followed by the novel combination of items 3, 4, and 9 (0.48) (Table 2). The highest internal consistency of the four-item combinations was obtained with the two established variations AUDIT-4 (0.67) and the FAST (0.66), followed by combination 3, 4, 8 and 9 (0.62).

### Variance explained

Of all three-item combinations, the combination of items 3, 4 and 9 explained the most variance (80.1%) in AUDIT-10 score while AUDIT-C explained only 55.8% of variance. Of the four-item combinations, the combination 3, 4, 7, and 9 explained the most variance (87.4%) in AUDIT-10, with numerous other four-item combinations explaining greater than 86% variance (Table 2). In comparison, AUDIT-4 explained 70.0% and the FAST explained 85.6% of variance in AUDIT-10 score.

### Pearson’s correlation coefficient (concurrent validity)

Correlation with the AUDIT-10 was highest with the three-item combination 3, 4 and 9 (0.88), but four other combinations demonstrated correlation of at least 0.85. In contrast, the correlation between AUDIT-C and AUDIT-10 was lower. Six variations of four-item combinations, including the FAST, had a correlation coefficient of 0.92 with AUDIT-10. In contrast, correlation between AUDIT-4 and AUDIT-10 was lower with a coefficient of only 0.83.

## Discussion

This study highlights how shortened versions of the AUDIT, using just a few items, can capture much of the information available from the full AUDIT scale, thus suggesting they may be as effective a screening tool to measure hazardous or harmful alcohol consumption. A number of shortened versions of the AUDIT performed well in relation to the AUDIT-10 by means of internal consistency, variance explained, and concurrent validity. Although some three-item combinations scored highest for individual measures, four-item combinations scored more consistently high across all statistical tests. Novel AUDIT versions may be more effective than many established shortened versions as an alternative screening tool to the AUDIT-10 to measure hazardous or harmful alcohol consumption in community-based populations of young Australians.

While AUDIT-C was the only AUDIT version under our study criteria which met the Cronbach’s alpha cut-off of at least 0.7 for research purposes [5,35], it did not perform well by other measures. The AUDIT-C exists in a single AUDIT domain based on consumption patterns, and thus items are more likely to correlate with each other, leading to a higher Cronbach’s alpha [36]. In contrast, the alternative novel versions crossed three domains that measured different types of alcohol misuse, as prioritised in the study protocol, so lower internal consistency may be reasonable.

Using just three of ten AUDIT questions was sufficient to explain over 80% of total variance in the AUDIT-10 score, with any additional items increasing this figure slightly. In general, correlation with AUDIT-10 was high, which may be due to the shortened AUDIT versions being subsets of the AUDIT-10 and not independent measures, as well as our study criteria for only considering novel combinations which represented all three AUDIT domains. In contrast, the AUDIT-C, which only covers one domain, explained considerably less variance and had lower correlation to the AUDIT-10 than novel combinations, which was also observed in a previous computerised survey of young people [37].

Our results suggest that numerous variations of the AUDIT scale may be a suitable alternative to the AUDIT-10 for classifying harmful and hazardous drinking in this sample of young people. When considering performance of AUDIT variations across all statistical measures, the FAST scored highest overall. However, the FAST does not include item 9 on alcohol-related injury, which was prioritised due to its significance to young people [33]. Nonetheless, there are advantages to using the FAST, given it has been validated in other settings, albeit primarily emergency departments [13,17,21]. After FAST, the two top-performing variations were similar: 3, 4, 8 and 9 or 3, 4, 5, and 9. There is no sound basis for choosing one of these combinations over the other in this sample population, although item 8 on memory loss has been shown to be a useful measure of harm and predictor of future alcohol-related injury in young people [37,38].

A number of limitations to this study and interpretation should be considered. The survey did not include a gold-standard or comparative clinical diagnosis of harmful/hazardous alcohol use or alcohol-related problems, such as DSM-IV, meaning we were unable to assess predictive validity. Subsequently, novel AUDIT variations were compared to the AUDIT-10, which itself has not been validated in this particular population, and some prior research has reported that the AUDIT is less reliable in the general population compared to clinical settings [8]. As such, we are only able to determine the performance of the shortened AUDIT variations in comparison to the full AUDIT, rather than as a predictor of harmful drinking *per se*; agreement does not infer accuracy of either the shortened versions or AUDIT-10 as a predictor of hazardous drinking or high-level drinking problems. Furthermore, the shortened AUDIT models are not independent of the AUDIT-10, and thus undermine the assumption of independence for assessing linear regression (variance explained). While this method has been used in previous related studies, it should still be interpreted with caution (e.g. [37]). A modified version of the FAST that was not gender-specific was used and may have overstated the performance of FAST in comparison to AUDIT-10.

This study population is based on a convenience sample and is not intended to be representative of all young Australians. In addition, due to the nature of the recruitment setting, we were unable to assess a response rate for participation. Prior surveys have demonstrated that participants recruited at the Melbourne Big Day Out music festival are more likely to engage in alcohol-, drug-, and sex-related risk behaviours than other young Australians [39,40]. While the high-risk nature of this sub-population makes them an important group for testing the performance of AUDIT variations, results may differ in other populations. Further research is needed to confirm findings in broader and more representative samples.

## Conclusion

Among a sample of young people commonly reporting risky alcohol consumption, we identified a number of novel three and four-item AUDIT variations, as well as the established FAST scale, which were suitable proxies of the AUDIT-10. Although it is difficult to ascertain a single standout shortened AUDIT variation in this population, numerous variations performed better than the more widely used AUDIT-C according to multiple criteria. Four-item combinations scored more consistently high across evaluated statistical measures and are the preferred compromise for maximising the indication of alcohol misuse while ensuring a short and simple measure of hazardous drinking in a community-based sample of young people.

## Competing interests

The authors report no competing interests relating to this manuscript.

## Authors’ contributions

The following co-authors have contributed to the work: AB in data collection, data analysis, manuscript preparation and manuscript review; MG in data analysis and manuscript review; MH in study design, manuscript preparation and manuscript review; and PD in study design, data analysis, manuscript preparation and manuscript review. All authors read and approved the final manuscript.

## Pre-publication history

The pre-publication history for this paper can be accessed here:

http://www.biomedcentral.com/1471-2458/13/301/prepub
